# A Comparative Evaluation of Marginal Leakage and Shear Bond Strength of Cention N, Resin-Modified Glass Ionomer Cement (RMGIC), and Conventional Glass Ionomer Cement (GIC): An In Vitro Study

**DOI:** 10.7759/cureus.98770

**Published:** 2025-12-08

**Authors:** Khushboo Singh, Debapriya Pradhan, Saurabh Tiwari, Raksha Thakur, Priyamvada Sharma, Devika Agrawal, Mahima Singh, Devshree Jawalikar, Delphina Michael Kapoor, Jyoti Priiya Kodimela

**Affiliations:** 1 Department of Pedodontics and Preventive Dentistry, Hitkarini Dental College and Hospital, Jabalpur, IND; 2 Department of Pediatric and Preventive Dentistry, Hitkarini Dental College and Hospital, Jabalpur, IND; 3 Department of Pediatric Dentistry, Surendera Dental College and Research Institute, Sri Ganganagar, IND; 4 Department of Dentistry, Clove Dental, Mumbai, IND

**Keywords:** kruskal-wallis, lsd post hoc, methylene blue, stereomicroscope, thermocycling

## Abstract

Aim: This study aimed to compare and evaluate the marginal leakage and dentin shear bond strength (SBS) of Cention N and resin-modified glass ionomer cement (RMGIC), and to compare them with conventional glass ionomer cement (GIC) (GC Fuji IX, GC Corporation, Tokyo, Japan).

Methods: For the evaluation of marginal leakage, 30 extracted premolars were randomly divided into three groups. Class I cavities were prepared, and the cavities were restored with Cention N, RMGIC, and GIC Type IX. After thermocycling, the specimens were immersed in 0.5% methylene blue dye for 24 hours. They were then sectioned and examined under a stereomicroscope at 40× magnification for scoring. Data were statistically analyzed using the Kruskal-Wallis and Mann-Whitney U tests. For dentin SBS evaluation, the occlusal surfaces of 30 premolars were ground flat, and cylinders of three restorative materials (n = 10 per group), Cention N, RMGIC, and GIC Type IX, were bonded to the surfaces following the manufacturers’ instructions. After 24 hours of storage at 100% humidity, dentin SBS was measured, and fracture modes were assessed under a stereomicroscope at 10× magnification. Data were statistically analyzed using the one-way ANOVA with the least significant difference post hoc test for SBS, and the chi-square test for fracture modes (p < 0.05).

Results: Cention N had the least marginal leakage and highest dentin SBS, followed by RMGIC and GIC type IX.

Conclusion: Cention N showed the least marginal leakage and highest dentin SBS, followed by RMGIC, while GIC Type IX performed the poorest. Thus, Cention N is recommended as a permanent restorative material for better clinical success and longevity.

## Introduction

The human tooth is a complex organ with a limited capacity for regeneration. The specialized tissues within it have a weak ability to heal themselves, making the preservation of vitality crucial. Therefore, replacing lost tooth structure is essential to maintain its form, functionality, esthetics, and long-term clinical durability [1]. For many years, dental amalgam has served as a trusted and commonly used material. However, its limitations, including mercury toxicity, higher coefficient of thermal expansion, and poor esthetics, prompted the development of alternative solutions. In 1972, Wilson and Kent introduced glass ionomer cement (GIC), which forms chemical adhesive bonds with tooth structure. Since then, GIC has gained widespread use due to its numerous advantages, such as moderate esthetics, radiopacity, biocompatibility, anticariogenic character due to fluoride release, lack of exothermic polymerization, excellent adhesion to dentin, chemical bonding, and the ability to remineralize adjacent dentin [1,2]. However, GIC is recommended for primary teeth, although its main disadvantages include low fracture toughness, poor wear resistance, and high solubility [1].

GC Fuji IX (GC Corporation, Tokyo, Japan), introduced in the late 1990s, was specifically designed for geriatric and pediatric patients. Known as a condensable or packable GIC, it offers enhanced strength, high viscosity, and improved esthetics. These advancements are attributed to the reduced size of glass particles in its matrix [2].

Resin-modified glass ionomer cement (RMGIC) was developed as an advanced material to overcome the limitations of conventional GIC. Designed to improve the physical properties of traditional GIC, RMGIC offers several advantages, including extended working time, faster setting through light curing, improved marginal sealing, fluoride release, enhanced esthetics, greater strength, and the chemical/mechanical bond to dentin. Its fluoride-releasing ability allows fluoride to bind directly to the tooth structure without requiring an additional bonding agent [3]. RMGICs bond to dentin through two primary mechanisms. The first is chemical adhesion, where the anions of polyacrylic acids interact with the calcium ions in the mineral apatite of dentin. The second is micromechanical retention. Moreover, the self-etching ability of RMGICs promotes the development of a shallow hybrid layer on conditioned dentin [4].

Cention N is a tooth-colored, posterior direct restorative material introduced as a "powder-liquid filling system." It belongs to the urethane dimethacrylate-based alkasite group, containing alkaline fillers that release acid-neutralizing ions. The addition of isofiller with low elasticity reduces shrinkage stress, which in turn decreases polymerization shrinkage and microleakage. Additionally, the alkaline glass fillers enable the release of fluoride, calcium, and hydroxide ions, which offer benefits, particularly in pediatric applications. Cention N is a dual-cure material that permits bulk placement, with or without the use of adhesives [1].

Strong adhesion of restorative materials to dentin enhances their retention in the oral cavity. The shear bond strength (SBS) of a material reflects its ability to withstand oblique forces, and higher SBS values indicate superior bonding between the restoration and tooth structure [5].

A strong marginal seal and enhanced bond strength are crucial for the longevity of restorative materials, as they help minimize marginal leakage, the primary cause of secondary caries, restoration staining, tooth discoloration, marginal deterioration, postoperative sensitivity, and pulpal pathology [6].

The clinical performance of newer restorative materials relies on strong adhesion to the dentinal surface, enabling resistance against dislodging forces [6]. Hence, the current study aimed to assess and compare two key parameters, marginal leakage and dentin SBS, of Cention N and RMGIC with those of GC Fuji IX, a conventional GIC. The null hypothesis proposed that there would be no significant difference in marginal leakage and dentin SBS among the three restorative materials evaluated.

## Materials and methods

The present in vitro study was carried out in the Departments of Pediatric and Preventive Dentistry at Hitkarini Dental College and Hospital, Jabalpur, after obtaining clearance and approval from the concerned Ethical Committee (HDC&H/2023/1175/N).

**Figure 1:**
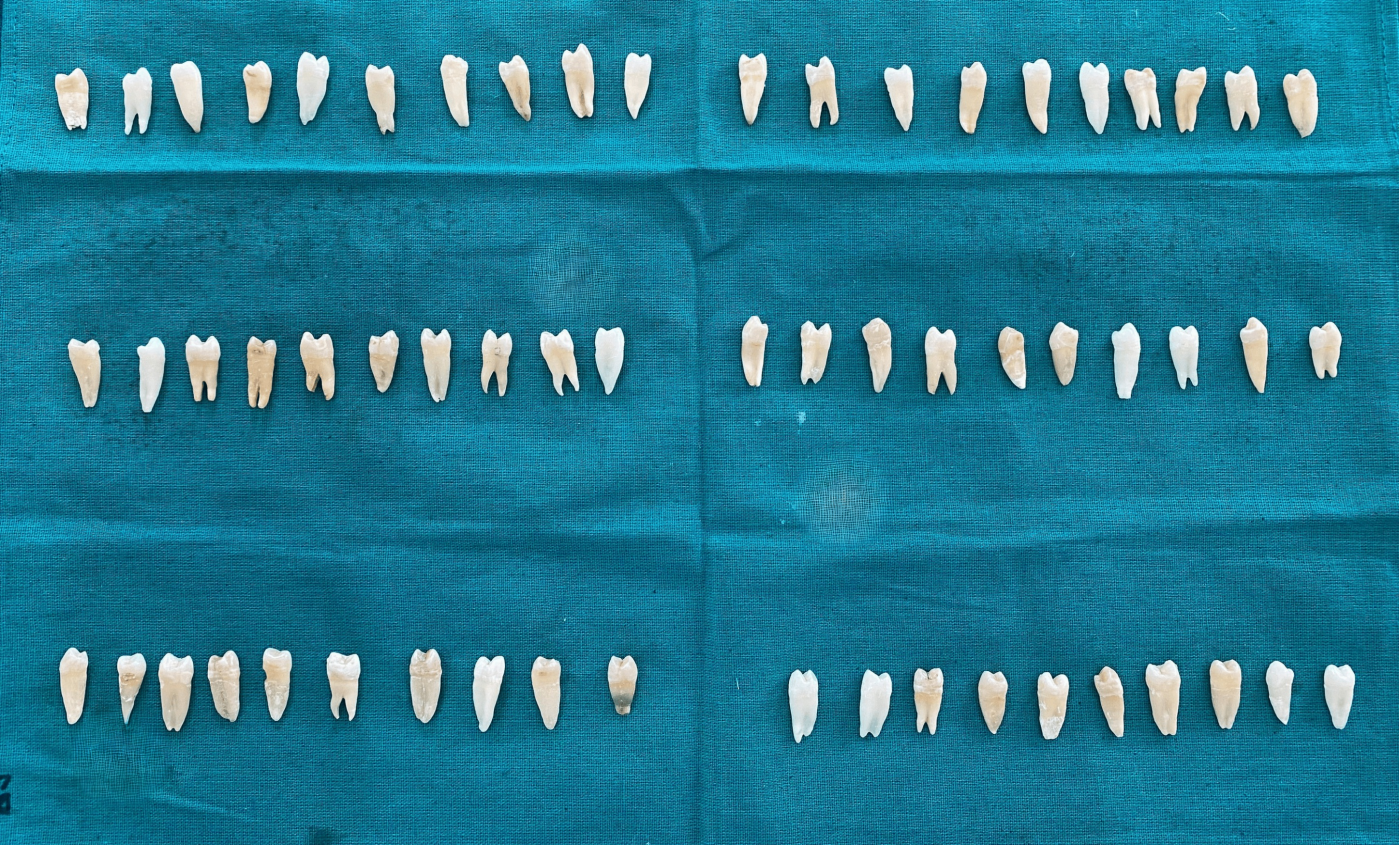
Total test specimens

The sample size was calculated using the G*Power version 3.1 software (Heinrich-Heine-Universität Düsseldorf, Düsseldorf, Germany), with an 80% power and a 5% level of significance. A total of 60 sound premolar teeth were extracted for orthodontic reasons from patients aged 12-18 years, with intact crowns, no caries, cracks, restorations, fluorosis, or developmental defects. Teeth with structural defects, previous restorative work, root resorption, or visible cracks under magnification were excluded. Each tooth was meticulously scaled to eliminate calculus and residual tissue, followed by polishing with a pumice slurry (Figure 1). The teeth were then stored in 0.9% saline at room temperature and used within three months of extraction, following Occupational Safety and Health Administration guidelines. The specimens were randomly divided into three groups (n = 10 per group) using a computer-generated random number sequence (Microsoft Excel; Microsoft Corporation, Redmond, WA) to eliminate selection bias (Table 1).

**Table 1:** Distribution of premolar teeth samples in study groups RMGIC: resin-modified glass ionomer cement; GIC: glass ionomer cement

Group	Material	Manufacturer	Marginal leakage	Shear bond strength	Total
Group I	Cention N	Ivoclar, Vivadent AG, Schaan/Liechtenstein	10	10	20
Group II	RMGIC	GC America	10	10	20
Group III	Type IX GIC	GC Gold Label, America	10	10	20
Total			30	30	60

A total of 60 premolar tooth samples were equally divided for testing marginal leakage and SBS.

Marginal leakage

**Figure 2:**
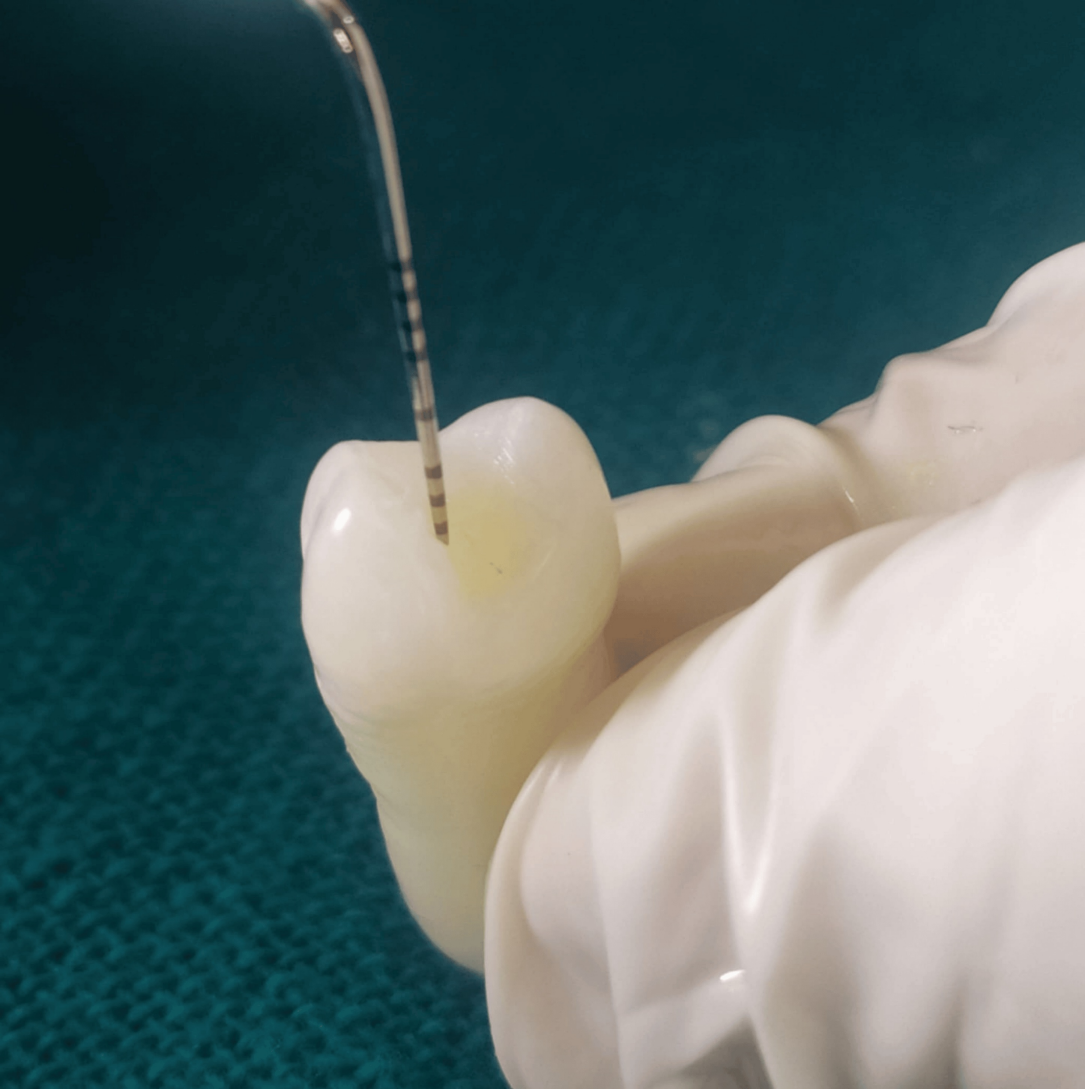
Cavity depth measurement by using a graduated probe

A standardized Class I cavity was created on the occlusal surface of 30 premolars using a No. 1 round bur with a high-speed airotor handpiece under continuous water cooling. The cavity dimensions were maintained at 3 mm in length, 2 mm in width, and 1.5 mm in depth, and standardized with the help of a divider, stainless steel scale, and graduated probe (Figure 2). Following preparation, the cavities were thoroughly cleaned, rinsed, and dried. The specimens were then randomly assigned to three groups, each consisting of 10 teeth.

Cention N was prepared according to the manufacturer’s guidelines, maintaining a powder-to-liquid ratio of 4.6:1 by weight. The material was placed into the cavity using a plastic filling instrument and light-cured with a visible light-curing unit (Woodpecker) for 20 seconds.

RMGIC was mixed following the manufacturer’s instructions with a powder-to-liquid ratio of 3.2:1, placed into the cavity using a plastic filling instrument, and light-cured using a Woodpecker curing unit for 20 seconds.

GC Fuji IX was prepared as per the manufacturer’s directions with a powder-to-liquid ratio of 3.6:1.0 g. The mixture was placed into the cavity using a plastic filling instrument, allowed to set for two minutes and 20 seconds, and then coated with petroleum jelly after a waiting period of five minutes. No adhesives or primers were used in any test group, and no additional conditioning was performed for any of the groups.

**Figure 3:**
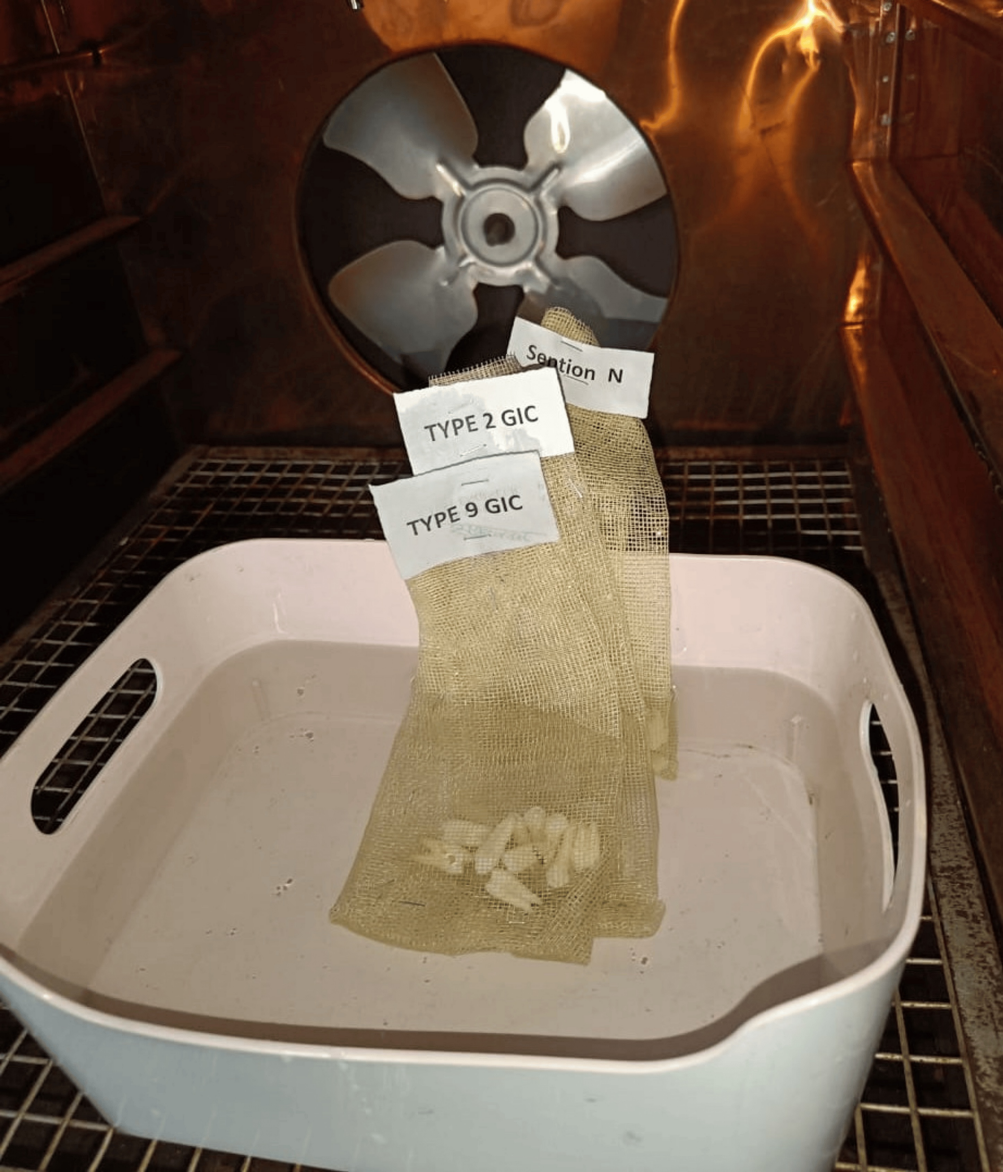
Thermocycling process

The samples were immersed in distilled water at room temperature for 24 hours before undergoing thermocycling. To simulate oral conditions, thermocycling was carried out according to ISO 11405 standards [7], subjecting all restored teeth to 500 cycles between 5°C and 55°C, with an immersion time of five seconds in each water bath (Figure 3).

**Figure 4:**
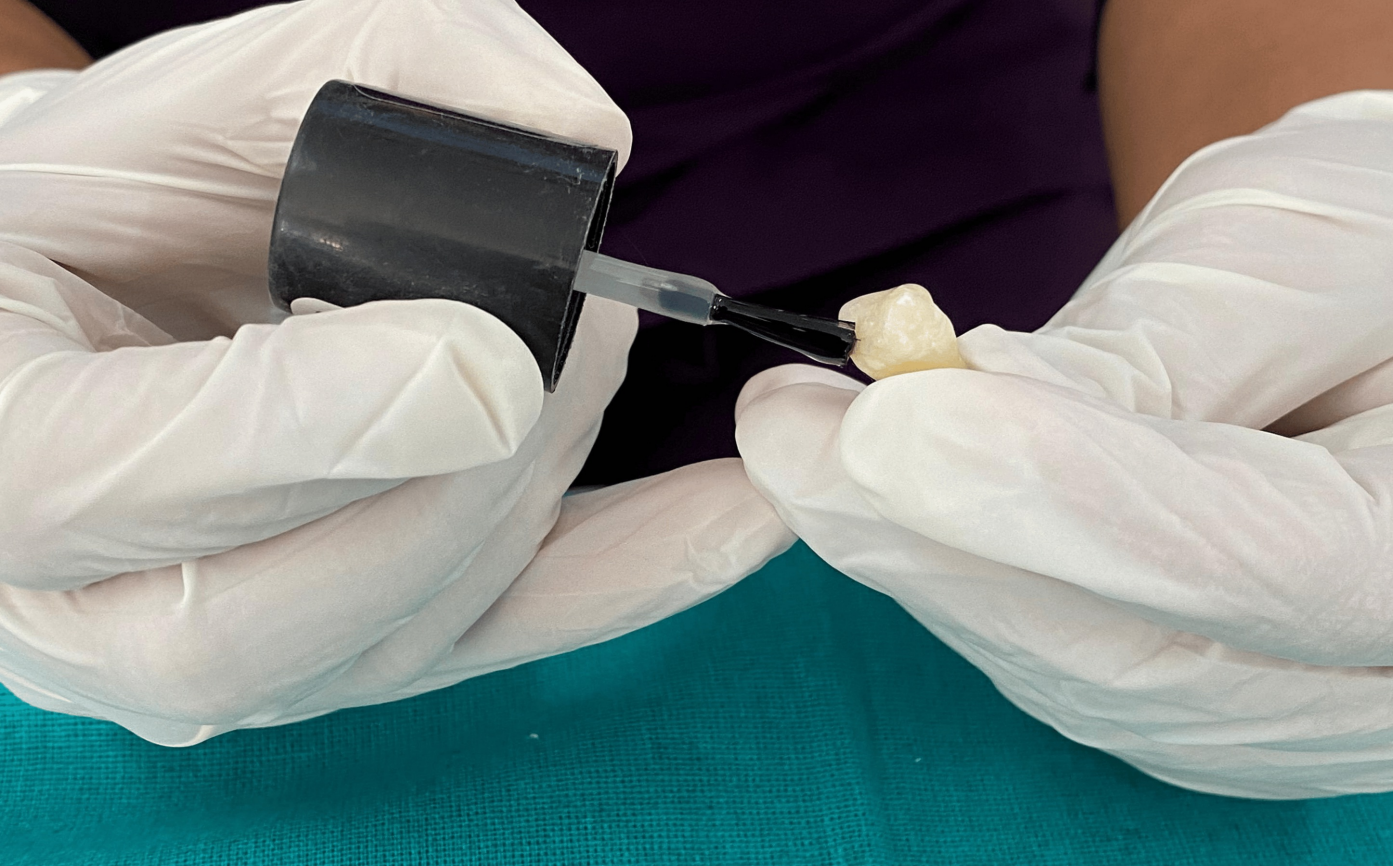
Nail varnish application

**Figure 5:**
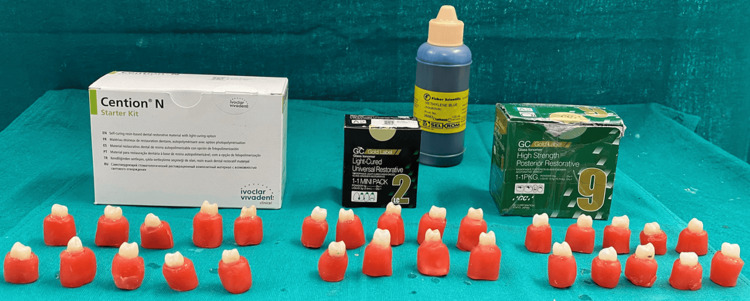
Sealing specimen apices with modeling wax

**Figure 6:**
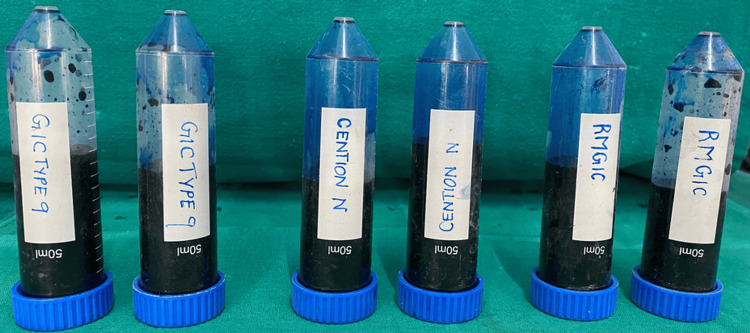
Sample in dye penetration

Following thermocycling, the specimens were prepared for dye penetration testing by coating them with fingernail varnish (Figure 4), leaving an uncoated margin of 0.5-1.0 mm around the restoration and sealing the apices with modeling wax (Figure 5). The samples were then immersed in 0.5% methylene blue dye for 24 hours (Figure 6) and subsequently rinsed thoroughly with water to eliminate any excess dye.

**Figure 7:**
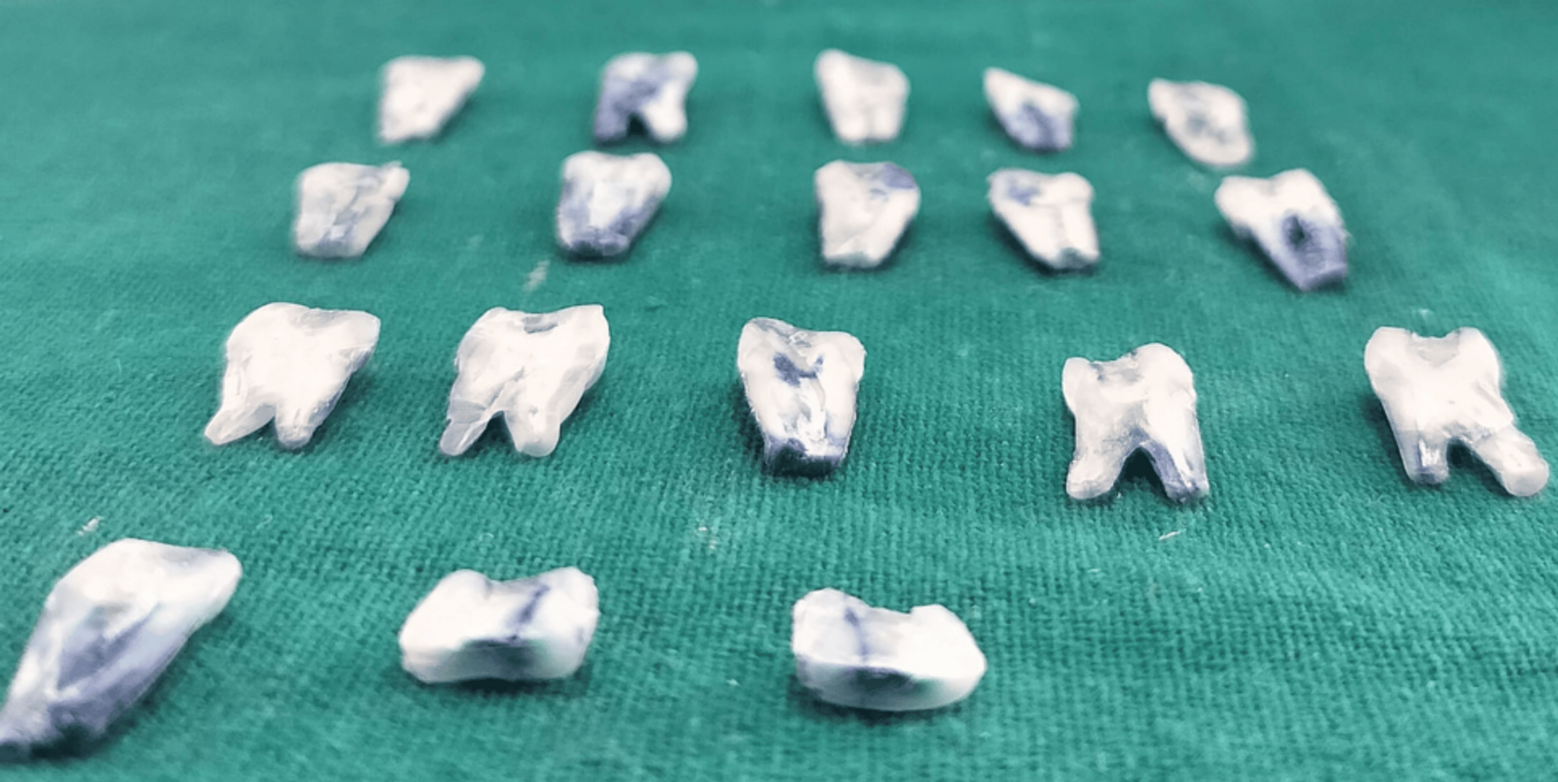
Tooth section for marginal leakage evaluation

Each specimen was sectioned longitudinally in a buccolingual direction through the center of the restoration using a micromotor fitted with a diamond disc (Figure 7). Microleakage was then assessed for all groups under a stereomicroscope at 40× magnification using the following scoring criteria [8]: 0: no dye penetration; 1: dye penetration between the restoration and the tooth into the enamel only; 2: penetration of dye at the interface between the restoration and the tooth structure within the enamel and dentin; and 3: dye penetration between the restoration and the tooth into the pulp chamber.

Dentin SBS

The occlusal surfaces of the remaining 30 premolars were flattened using a high-speed diamond disc under continuous water cooling, producing cuts perpendicular to the tooth’s longitudinal axis. The exposed dentin surfaces were then smoothed with 600-grit silicon carbide paper for 60 seconds under continuous water irrigation. All specimens were stored in distilled water at room temperature until their roots were embedded in self-polymerizing acrylic resin within silicone molds measuring 25 × 25 mm, leaving the coronal portions exposed above the resin blocks.

The embedded teeth were randomly divided into three groups of 10 specimens each. A double-sided adhesive tape with a 3 mm diameter punched hole was affixed to the dentin surface of each specimen to delineate a standardized bonding area. A plastic mold with an internal diameter of 3 mm and a height of 5 mm was positioned at the center of the exposed dentin surface. Each restorative material was prepared as previously described and inserted into the mold using a plastic spatula. GC Fuji IX was allowed to set for two minutes and 20 seconds, whereas RMGIC and Cention N were light-cured with a visible light-curing unit (Woodpecker) for 20 seconds to achieve immediate setting.

**Figure 8:**
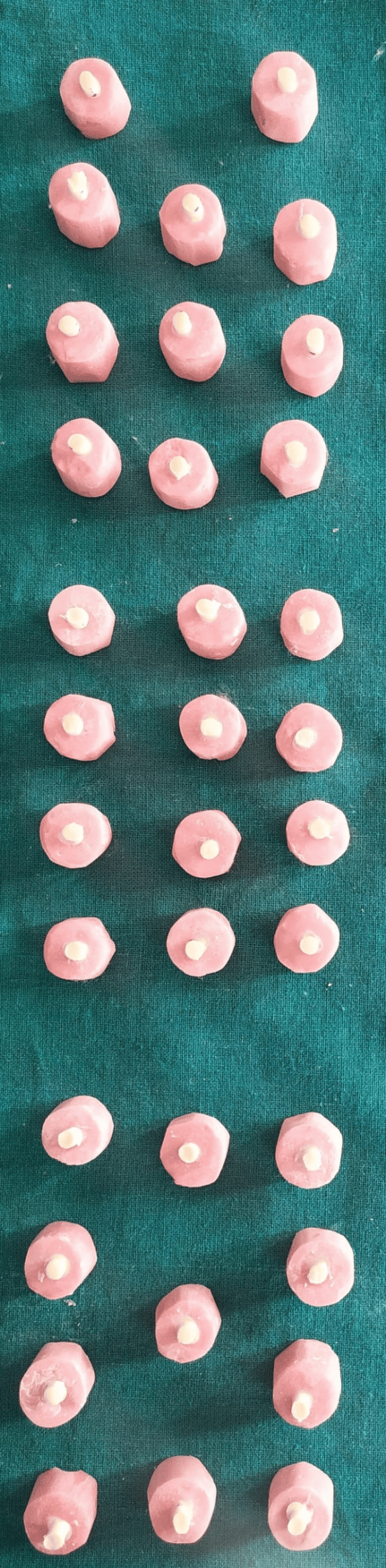
Restorative material specimen blocks

After ensuring complete setting of the restorative materials (approximately six to seven minutes), the mold was gently removed by making two parallel incisions with a Bard-Parker blade, ensuring the restoration remained undisturbed (Figure 8).

**Figure 9:**
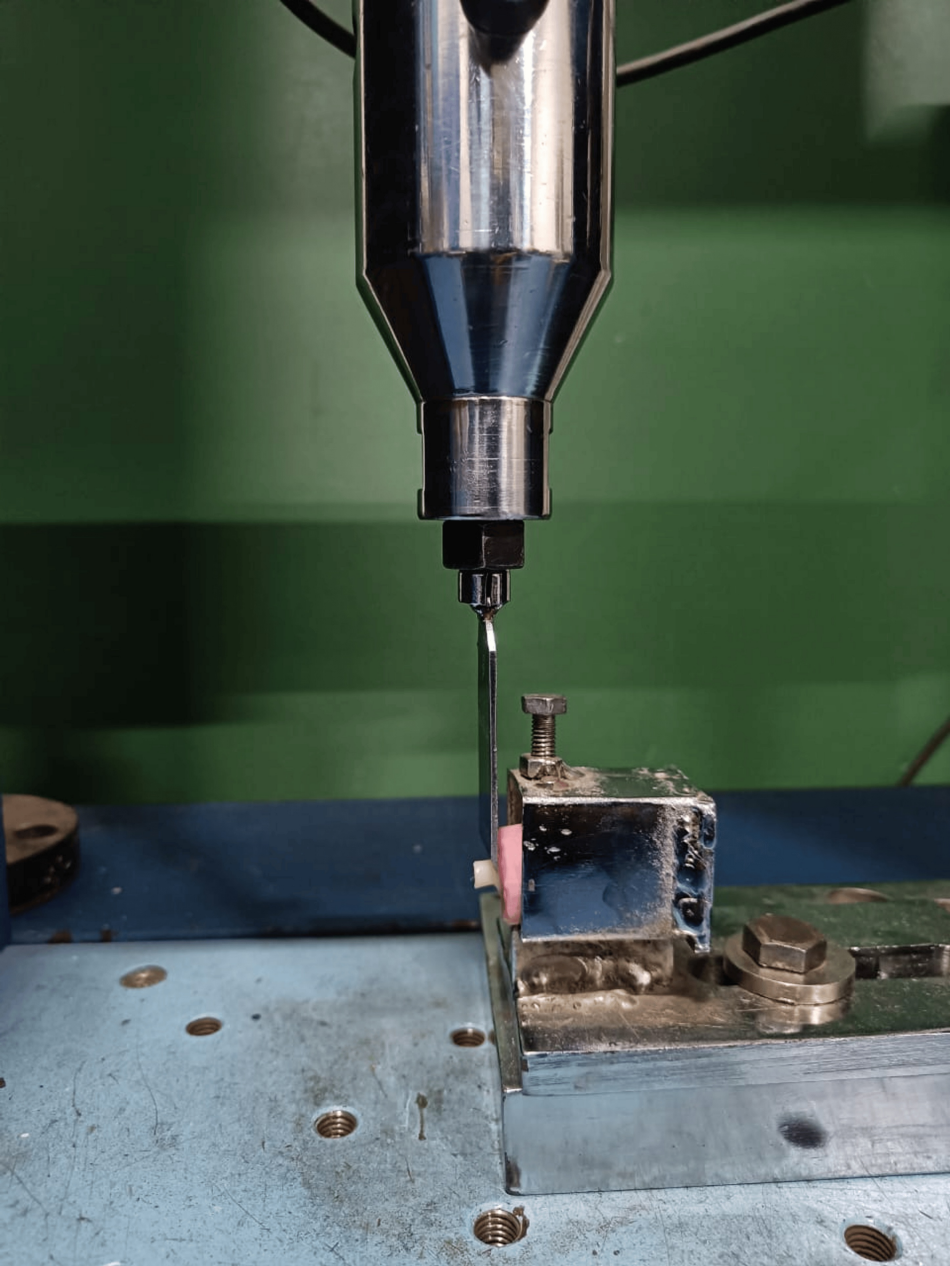
Shear bond strength testing under a universal testing machine

The specimens were placed in an incubator maintained at 37°C and 100% humidity for 24 hours [1]. Each specimen was then mounted on a computerized, software-based Universal Testing Machine (ACME Engineers, India), ensuring the dentin surface was aligned parallel to the loading device. A shear force was applied using a steel knife-edge positioned precisely at the dentin-restoration interface (Figure 9). The load was applied at a crosshead speed of 0.5 mm/minute until the restoration failed.

The force (N) required to dislodge the restoration was recorded, and the bond strength (MPa) was calculated by dividing the applied shear force by the bonding area (mm²). Following the shear test, the tooth specimens were examined under a stereomicroscope at 10× magnification to analyze the mode of fracture. Fracture modes were classified as follows [1]: adhesive (failure occurring at the junction between the restoration and the dentin), cohesive (failure within the restorative material), mixed (partly adhesive and partly cohesive fracture), and all the procedures were carried out by one investigator.

Statistical analysis

Data was entered in Microsoft Excel 365 for Windows. Frequencies, percentages, mean, standard deviation, and minimum and maximum values of variables were calculated. Descriptive statistics were calculated. Further analyses included Kruskal-Wallis and Mann-Whitney U tests for microleakage, one-way analysis of variance (ANOVA) with least significant difference post hoc for SBS, and chi-square tests for fracture modes (with Yates’ correction when expected cell frequency <5). The significance level was set at p < 0.05 for main effects. To control for Type I error during multiple pairwise comparisons (three pairs), a Bonferroni correction was applied, setting the significance threshold at p < 0.017 (0.05/3). Data analyses were performed using version 23.0 of the Statistical Package for Social Sciences (IBM Corporation, Armonk, New York).

## Results

Marginal leakage

Ten teeth from each group were evaluated for marginal leakage using a stereomicroscope. In the Cention N group, eight samples showed no dye penetration and were given a score of 0, while two samples showed dye penetration up to the enamel and were assigned a score of 1. In the RMGIC group, six samples showed no dye penetration (score 0), two samples showed dye penetration up to the enamel (score 1), one sample showed dye penetration up to the dentin (score 2), and one sample showed dye penetration up to the pulp chamber (score 3). In the Type IX GIC group, one sample showed no dye penetration (score 0), three samples showed dye penetration up to the enamel (score 1), one sample showed dye penetration up to the dentin (score 2), and five samples showed dye penetration up to the pulp chamber (score 3), as presented in Table 2.

**Table 2:** Distribution of marginal leakage scores in study groups RMGIC: resin-modified glass ionomer cement; GIC: glass ionomer cement

Groups	Marginal leakage score, n (%)				Total, n (%)
	Score 0	Score 1	Score 2	Score 3	
Cention N	8 (80)	2 (20)	0 (0)	0 (0)	10 (100)
RMGIC	6 (60)	2 (20)	1 (10)	1 (10)	10 (100)
Type IX GIC	1 (10)	3 (30)	1 (10)	5 (50)	10 (100)

Marginal leakage scores in the Type IX GIC group were significantly higher than the Cention N group (U = 9.000, p < 0.01), demonstrating a large effect size (r = 0.74). There was no significant difference for microleakage scores between Cention N and RMGIC groups (U = 38.000, p > 0.017), with a small effect size observed (r = 0.25). Also, there was no significant difference for microleakage scores between RMGIC and Type IX GIC groups (U = 20.000, p > 0.017). However, the analysis indicated a large effect size (r = 0.53) for this comparison (Table 3).

**Table 3:** Comparison of marginal leakage scores between study groups Kruskal-Wallis test: χ2 = 12.337, df = 2, p = 0.002 (<0.01) is significant Mann-Whitney U test: Cention N and RMGIC: U = 38.000, r = 0.25, p = 0.261 (>0.017) is not significant; Cention N and Type IX GIC: U = 9.000, r = 0.74, p = 0.001 (<0.01) is significant; RMGIC and Type IX GIC: U = 20.000, r = 0.53, p = 0.018 (>0.017) is not significant RMGIC: resin-modified glass ionomer cement; GIC: glass ionomer cement; SD: standard deviation

Groups	Marginal leakage score	
	Mean ± SD	Min-Max
Cention N	0.20 ± 0.42	0-1
RMGIC	0.70 ± 1.06	0-3
Type IX GIC	2.00 ± 1.16	0-3

Dentin SBS

Ten samples from each group were tested for Dentin SBS, and the values were recorded. Cention N showed the highest SBS, followed by RMGIC, and the least with conventional GIC (Type IX GIC) (Table 4).

**Table 4:** Comparison of shear bond strength between the study groups Welch’s one-way ANOVA: F (2, 13.51) = 137.385, p = 0.000 (<0.001) is significant Games-Howell post hoc test: Cention N and RMGIC: mean difference: 0.52, 95% CI (-0.43 to 1.48), p = 0.356 (>0.017) is not significant; Cention N and Type IX GIC: mean difference: 3.46, 95% CI (2.60-4.32), p = 0.000 (<0.001) is significant; RMGIC and Type IX GIC: mean difference: 2.94, 95% CI (2.35-3.53), p = 0.000 (<0.001) is significant RMGIC: resin-modified glass ionomer cement; GIC: glass ionomer cement; SD: standard deviation; ANOVA: analysis of variance; CI: confidence interval

Groups	Shear bond strength (MPa)	
	Mean ± SD	Min-Max
Cention N	4.13 ± 0.96	2.34-5.85
RMGIC	3.61 ± 0.66	2.59-4.58
Type IX GIC	0.67 ± 0.20	0.40-1.06

Table 4 compares SBS between the study groups. The mean ± SD of SBSs in Cention N, RMGIC and Type IX GIC groups were 4.13 ± 0.96, 3.61 ± 0.66, and 0.67 ± 0.20 MPa, respectively. Minimum and maximum values of SBSs in Cention N group were 2.34 and 5.85 MPa, in RMGIC were 2.59 and 4.58 MPa and in Type IX GIC groups were 0.40 and 1.06 MPa. Welch’s one-way ANOVA showed a significant difference among the groups for SBSs (F(2, 13.51) = 137.385, p < 0.001). After the significant result of one-way Welch’s one-way ANOVA, the Games-Howell post hoc test (adjusted α = 0.017) was applied for pairwise comparison, which showed the following observations: 1) SBSs in Type IX GIC group were significantly lower than Cention N and RMGIC groups (mean difference: 3.46 MPa; 95% CI, 2.60-4.32; p <0.001 and mean difference: 2.94 MPa; 95% CI, 2.35-3.53; p < 0.001, respectively), and 2) there was no significant difference for SBSs between Cention N and RMGIC groups (mean difference: 0.52 MPa; 95% CI, -0.43 to 1.48; p > 0.017).

According to the failure mode analysis, GC Fuji IX showed predominantly cohesive failures, RMGIC exhibited mostly mixed failures, whereas Cention N demonstrated primarily adhesive failures (Table 5).

**Table 5:** Comparison of fracture modes between study groups Chi-square test: Yates’ χ2 = 16.407, df = 4, p = 0.003 (<0.01) is highly significant RMGIC: resin-modified glass ionomer cement; GIC: glass ionomer cement

Groups	Fracture modes, n (%)			Total, n (%)
	Adhesive	Cohesive	Mixed	
N	7 (70)	1 (10)	2 (20)	10 (100)
RMGIC	2 (20)	1 (10)	7 (70)	10 (100)
Type IX GIC	0 (0)	8 (80)	2 (20)	10 (100)

## Discussion

The fundamental purpose of a dental restorative material is to reestablish the natural function, appearance, and biological integrity of the tooth structure. Stronger restorative materials help resist fracture and deformation, ensure uniform stress distribution, enhance stability, and ultimately improve clinical success [2].

The present study assessed the marginal leakage, Dentin SBS, and fracture mode analysis of two modified restorative materials, Cention N and RMGIC and compared their performance with GC Fuji IX, a conventional GIC. The null hypothesis was rejected because significant differences were observed in marginal leakage, dentin bond strength, and fracture mode analysis among the three materials.

In this study, the commonly used 0.5% methylene blue dye method was employed to evaluate marginal leakage after restoring Class I cavities on the occlusal surfaces of human premolars. The occlusal surfaces are more prone to caries, and occlusal cavities can be easily standardized. The dye molecules, measuring 0.80 nm in diameter, are smaller than the dentinal tubules. The permeability of dentinal tubules, combined with the dye's small particle size, may lead to an overestimation of its penetration significance. The same dye was employed in the studies conducted by Kumari and Singh [1] and Raju et al. [6]. To replicate the thermal changes occurring in the oral cavity, thermocycling was performed.

The present study revealed that Cention N exhibited the least microleakage (0.20 ± 0.42) (Table 3), compared with RMGIC (0.70 ± 1.06) and GC Fuji IX (2.00 ± 1.16). Similar findings were reported by Dennis et al. [9] and Iftikhar et al. [10]. This may be attributed to the high polymer network density of Cention N, which allows polymerization throughout the entire depth of the restoration. Its patented isofiller, partially silanized, acts like a spring during polymerization, expanding to push the material against cavity walls, improving adaptation and reducing marginal leakage.

Although the comparison between RMGIC and Type IX GIC showed a large effect size (r = 0.53), it did not reach statistical significance after correcting for multiple comparisons (p = 0.018). This suggests a potential clinical difference that requires a larger sample size to confirm. Similar findings were reported by Pavuluri et al., who found no significant difference in microleakage between RMGIC and conventional GIC [8]. However, not all studies support this result; for instance, Singla et al. and Khan et al. found a significant difference in microleakage between RMGIC and conventional GIC [11,12]. The lower microleakage scores of RMGIC compared to GC Fuji IX may be attributed to its superior sealing ability due to efficient sealing ability of light-cured, resin-reinforced restorative cement, which may be explained by water sorption, a property of the resin components, which could have caused expansion of the material and consequently reduced the marginal gaps between the restoration and the tooth [13]. The highest microleakage observed with GC Fuji IX may be attributed to its high viscosity, which hinders proper wetting of the tooth surface and prevents the formation of an effective seal at the tooth-restoration [14].

Assessment of dentin SBS was another focus of the present in vitro investigation. A high bond strength in restorative materials enhances their ability to withstand various dislodging forces during function [1]. Dentin SBS values were highest for Cention N, followed in order by RMGIC and GIC type IX. The results are consistent with the findings of Chandra et al., who documented the highest mean bond strength for Cention N and the lowest for GC Fuji IX [2]. The higher SBS of Cention N supports the findings of Mehra et al., who reported significantly greater values for Cention N than for RMGIC when bonded to composite [15]. Somani et al. reported that the SBS of RMGIC to dentin is superior to that of conventional GC Fuji IX [16].

Evaluating bond failure provides insights into the nature of adhesion between restorative materials and tooth structure. According to the results of our study, Table 5 illustrates the distribution of different failure modes among the three materials tested. Cention N exhibited seven adhesive failures, two mixed failures, and one cohesive failure. In the case of RMGIC, two adhesive, seven mixed, and one cohesive failure were recorded. Conventional GIC showed no adhesive failures, with eight cohesive and two mixed failures. Overall, cohesive failure was predominantly associated with conventional glass ionomer, mixed failures were most common with RMGIC, whereas adhesive failure occurred most frequently with Cention N. This observation is consistent with the findings of Kumari and Singh [1] and Feiz et al. [17], indicating a rupture at the interface between the tooth and the restoration. According to the results of our study and Ugurlu, the high incidence of mixed and cohesive failure types in RMGIC could be attributed to voids formed by air entrapment during hand mixing [18]. Similarly, Murthy and Murthy observed that the brittle nature of GIC typically results in cohesive failure rather than failure within the ion-exchange layer [19].

Limitations of the study

As this investigation was conducted in vitro, the clinical behavior of the tested materials cannot be accurately anticipated based solely on these findings. Well-designed clinical trials are essential to establish definitive conclusions regarding the microleakage and dentin SBS of various restorative materials. Cention N is a relatively new restorative material, and limited literature is currently available on its performance. Therefore, further research and clinical evaluations are needed to substantiate its efficacy.

## Conclusions

Within the confines of the present in vitro study, it can be concluded that Cention N showed the least microleakage and highest dentin SBS, followed by RMGIC, while GIC Type IX performed the poorest. Thus, Cention N is recommended as a permanent restorative material for better clinical success and longevity.
